# Can nonpartisan primaries boost turnout and lessen demographic disparities?

**DOI:** 10.1371/journal.pone.0335840

**Published:** 2025-12-08

**Authors:** Todd Donovan, Nathan K. Micatka, Caroline J. Tolbert

**Affiliations:** 1 Department of Political Science, Western Washington University, Bellingham, Washington, United States of America; 2 Department of Political Science and Criminal Justice, University of South Alabama, Mobile, Alabama, United States of America; 3 Department of Political Science, University of Iowa, Iowa City, Iowa, United States of America; The University of Warwick, UNITED KINGDOM OF GREAT BRITAIN AND NORTHERN IRELAND

## Abstract

U.S. congressional general elections are largely uncompetitive, featuring candidates who emerge from low-turnout primaries. Voters in these primaries are unrepresentative of the broader electorate; primary voters tend to be older and wealthier. Previous research has focused on how open and nonpartisan primaries may affect the turnout of unaffiliated voters in primaries. We are interested in the downstream effects of opening primaries, including their estimated effects on turnout of younger voters and those with lower socioeconomic status. This study uses large samples of administrative voter file data to investigate whether nonpartisan and open primaries were associated with higher turnout across multiple elections (2018 and 2022). Results find open primaries were not associated with higher individual-level turnout. We found that nonpartisan primaries were associated with higher turnout across all age groups, including both low- and high-income groups, as well as low- and high-education groups. The results find the greatest proportionate increases for younger voters.

## Introduction

Congressional primaries serve as first-round elections to select candidates appearing on the general election ballot. Turnout in midterm primary elections is low, averaging 20 percent of eligible voters nationwide [1]. Since most congressional elections are not competitive due to gerrymandered districts and geographic self-sorting [2], primaries represent one of the few competitive elections for Congress or state legislatures [3]. One potential problem is that most people do not vote in primaries, and those who do tend to be older and more affluent [4] – such elections may produce biased representation. This study examines how the choice of state primary rules (closed, partially open, open, or nonpartisan) affects turnout among different demographic groups defined by age, income, and education in congressional primaries. Unlike previous research that relied on aggregate turnout data for states or self-reported turnout from surveys with sampling bias and the potential for overreporting voting, we rely on state voter file data and commercial data used to estimate some demographic traits for all US adults to present a more accurate picture of who votes in primary elections and how election laws affect participation rates.

A key question motivating this study is how primaries that are open to unaffiliated voters or nonpartisan primaries may have differentially higher turnout across demographic groups and thus potentially have more representative electorates [5–7]. In most states’ primaries, Republican candidates compete against other Republicans, while Democratic candidates compete against each other. In states with closed primaries, only registered party members vote, and strong partisans are the most likely to participate [8]. Conversely, in states with open or partially-open primaries, independents are allowed to participate. Because these individuals make up a third of the US population, electorates under these systems are more representative. In just four states with nonpartisan or all-candidate primaries, candidates from the Democrats, Republicans, and independents compete against one another in the same primary open to all voters. Some contend that differences between closed and open primaries are minor, and rule changes would not likely affect turnout [9–10]. Nonetheless, open primaries have high support among the mass public, with roughly 3 in 4 US adults approving of allowing unaffiliated people to vote in primaries [11]. Since unaffiliated voters are disproportionately young, less affluent, and people of color [12], this study is interested in the possible downstream effects of open primaries on the turnout of various demographic groups.

### The primary ‘problem’ & possible solutions

Research finds that closed primary rules that only allow registered party members to participate exclude over 27 million unaffiliated registered voters [13]. These authors refer to this as the “primary problem.” Some states have adopted open or nonpartisan primary rules, which permit individuals of any or no party alignment to participate in these elections. Barriers to primary participation for unaffiliated voters posed by closed primary rules may be further exacerbated by other trends in American politics, including partisan gerrymandering and geographic self-sorting of the electorate along ideological lines. This makes most House elections in the U.S. uncompetitive, with primaries determining outcomes. The Cook Political Report notes only 22 of 435 House races were a toss-up in 2024, or 5 percent. Nearly 95 percent of House seats are safe for one political party or the other (Cook Political Report, 270towin.com). In 2022, a mere 8 percent of voters elected 83 percent of the U.S. House of Representatives in uncompetitive general election districts (Unite America Institute report, ivn.us). Thus, a potential problem with the representativeness of Congress may be a lack of influence in primaries of unaffiliated voters, including those who may be younger and less affluent.

The 2024 American National Election Study (ANES) found 40% of people under 35 identified as independent (with 14.3% pure independents), compared to 29% of those 65 and older (with 10.5% pure independent); and 38.7% of respondents with family income under $30,000 identifying independent, compared to 31.4 of people with family income over $100,000.

The Bipartisan Policy Center reports there has been a decline in the use of closed-party primaries and a growth in primaries open to unaffiliated voters. Over the past two decades, primaries open to unaffiliated voters have become more common, increasing from 17% to 25% of all primary elections [14]. Recently, however, some Republican states are moving towards closing their primaries, such as Wyoming. Yet, even where these open and nonpartisan primary rules have been adopted, the evidence of effects on turnout is mixed, with some studies showing minimal effects linked to open and nonpartisan systems and others finding higher turnout associated with them [3,15].

**Fig 1:**
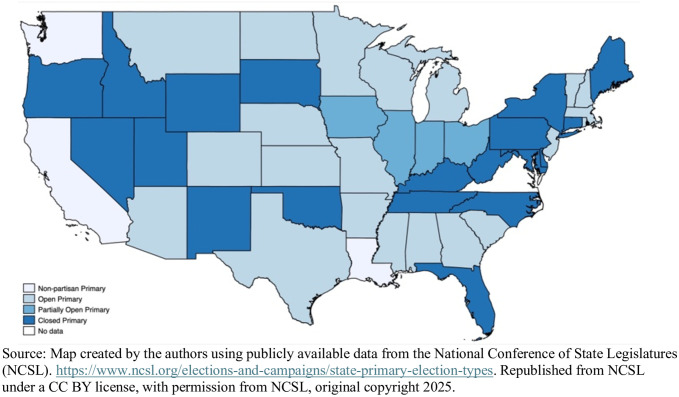
State primary election types using 2021 NCSL data.

Fig 1, created by the authors, is developed using NCSL data on state primary types. State primary laws have undergone significant changes over time. Our analysis relies on NCSL’s (2021) categorization of primary rules, focusing on primaries for congressional and state legislatures, not presidential primaries. For the statistical analysis and the map shown in Fig 1, we combine closed and partially closed as one category. Nineteen states have closed or partially closed primaries, including states in every region of the country and with Republican and Democratic party control of the state government. These states are shaded dark blue.

Only four states have nonpartisan primaries where candidates from both parties appear on one primary ballot, including Alaska, California, Louisiana, and Washington (note that Alaska is not shown in Fig 1). For the years considered in our analysis, Louisiana’s ‘primary’ was held in synch with November general elections and would produce a majority winner, so we exclude that state from our analysis. Twenty-two states have open primaries or primaries open to unaffiliated voters, most recently adopted in Colorado. These states are shaded light blue on the map. Four Midwest states (Illinois, Indiana, Iowa, and Ohio) have partially open primaries where independents can register as party members to vote in the primary on election day, but they lose their unaffiliated status. In the other open states, like Colorado, independents do not become registered with a party by voting in a party primary.

Some studies have found that states allowing unaffiliated voters to participate in primaries have higher turnout. Average aggregate state turnout is 5–6 percentage points higher when states open their primaries to unaffiliated voters via the nonpartisan primary [14,16]. Residents of states with nonpartisan primaries have a 12-percentage point higher probability of voting in midterm primaries, all else equal, compared to those living in closed primary states [15].

NCSL codes Nebraska (NE) as top-two for state legislative primaries, but NE is open partisan for congressional. Louisiana (LA) is unlike other nonpartisan primaries in that the majority winners were elected in a November ‘primary,’ or a December runoff. For our analysis of turnout in midterm primaries, NE is coded partisan (open), LA is omitted, and Virginia (VA) is also omitted, given that gubernatorial contests are in odd years and primaries are optional. Including VA does not change the results reported here.

### Do open primaries make electorates more demographically representative?

A Bipartisan Policy Center, October 2024 report using voter file data from the commercial vendor L2, suggests that primary voters are not representative of the pool of eligible US adults or voters in general elections [8,14] Unaffiliated voters or people who were not registered with a political party were estimated to constitute nearly 1 in 3 (28%) of the average state’s eligible voter population but only 23% of the state’s general electorate and only 10% of primary electorates. Nine in 10 primary voters are registered Republicans or Democrats, even though self-identified independents are a plurality of US adults (see Gallup’s historical partisanship trends, news.gallup.com).

Some demographic groups may be more likely to vote in primaries than in general elections. Average state primary electorates have been found to be skewed towards older people [14] Those authors also found low-income voters are somewhat underrepresented in the primaries. Their analysis finds that open primaries, primaries open to unaffiliated voters, and nonpartisan primaries tend to result in primary electorates that better represent the population of *independent voters* in states. However, state primary rules were not found to be related to the electorate’s composition across racial demographics, age or gender, income, or veteran status according to primary type.

Our study examines primary turnout for demographic groups using different commercial data (Catalist) that, we suggest, provides more accurate information on all voters (registered or not), and thus provides more accurate measures of individual-level citizen voting age population turnout, compared to voter file data that only includes registered voters.

A recent study of the demographic representation of Alaska’s primary electorate before and after use of a nonpartisan primary shows some potential effects of changed primary rules on the composition of the primary electorate. Although changes to various US election laws often fail to alter the demographic representation of electorates [17–18], advocates of Alaska’s change to a nonpartisan, top-four primary in 2022 had suggested that the partisanship/ideology of the participating primary electorate would become more representative of the state’s electorate if independent and moderate voters were allowed to participate. Using large samples of voters from state voter files combined with commercial data over time from the vendor Catalist, the study found primary turnout increased in 2022, proportionately the most among political independents, liberals, younger voters, and, to some extent, moderates [6]. Notably, young people, not just unaffiliated voters, were significantly more likely to participate under the nonpartisan primary than the state’s 2018 closed party primary. Given that young people are the least likely to vote and that nonpartisan primaries may lower barriers to participating in primaries, we expect younger people may have a higher likelihood of voting in nonpartisan primaries than closed primaries.

### Research design and data

One limitation of the relatively sparse research on the effects of open primaries is that previous research emphasizes the participation of unaffiliated voters rather than the demographic composition of electorates, with some exceptions. Additionally, while the voter file data used in by the Bipartisan Policy Center is a significant step forward in overcoming the limitations of survey data to study primary voters, the L2 vendor data includes only registered voters, has extensive missing data for some demographic variables like education and income, and has been shown to be less accurate when validated against survey data. Catalist includes registered voters and unregistered voting-aged adults, with demographic data on individuals that is not available with Census data or from other sources.

This study utilizes voter file data from the 50 states, combined with commercial data, to provide information on registered and unregistered individuals in the 2018 and 2022 midterm primaries, which represents the near-universe of the voting-eligible population in each state. The data are a random 1% sample of all 265 million US adults, with two roughly million cases for analysis. The data are from the vendor Catalist, one of the best data sources available for information on who votes in primary elections, as it is not susceptible to sampling or nonresponse bias. The national voter file combines official voting records from state voter files with additional data (e.g., U.S. Postal Service National Change of Address data), industry data (cell phone records, credit bureau reports), and campaign canvass records for the near universe of the adult U.S. population (see catalist.us/data).

Catalist has advantages in terms of breadth of coverage of nonregistered people 18 years or older as it identifies unregistered voters using commercial data, tracks registered voters who fall out of voter files, and tracks unregistered people who move states without registering [19]. There are some limitations, however. First, these data do not allow us to completely distinguish ineligible unregistered people (e.g., non-citizens, felons in states where they are ineligible to register) from people eligible to register to vote. Second, one study of the voter files estimated the unregistered population may be biased as it can miss 11% of US adults – disproportionately minority populations [20]. However, Table A7 in the Supplemental Information (S2 File) demonstrates that the overall primary turnout estimated using the Catalist individual-level data is a near match (less than a 1% difference) to the aggregate primary turnout. We also assume that potential bias in underestimating the ineligible population is minimized as it would take substantial short-term shifts of this population across states for this to consequently affect our estimates. This is an even smaller difference than found in an earlier study that concluded that voter file data provided by Catalist “do not suffer from any widespread lack of coverage” [21]

We measure if people defined as low, average, or high income, are more likely to vote in primaries depending on the primary type in their states, compared to all non-primary voters (voter age population). The analysis does the same for individuals from low- and high-education groups as well as young versus older people. The main explanatory variable is whether the individual lives in a state with open, partially open or nonpartisan primaries compared to states with closed primaries; the outcome variable is individual turnout in congressional primary elections.

Because survey data for low turnout primaries is sparse, and the data that is available is subject to random sampling error and overreporting turnout [22], the analysis reported in this study is based on individual turnout records from voter file data from the vendor Catalist. It allows measurement of who votes in elections compared to nonvoters, as the sample is more complete than relying on only registered voters. A growing number of studies have used Catalist data to study voter turnout and representation in the United States [6,19,23–27].

We analyze turnout data for 2018 and 2022 primary elections using a random 1% dataset drawn in October 2023. Importantly, Catalist comprehensively measures individual income using credit bureau reports (nearly all records have income data). We also analyze a probabilistic calculation of whether the respondent has a college degree or higher using administrative records provided in the data. Age is generally measured as part of the state voter file records from the birthdate. Validation against the 2022 Collaborative Midterm Survey (CMS) matched with Catalist data finds high accuracy for demographic factors, including family income (see Supplemental Information Tables A8 & A9 (S2 File)).

For the demographic subgroups, the statistical models predict voting in the 2022 primaries, using the person’s turnout in the 2018 primaries as a lagged variable. The models predict turnout of voting-age adults relative to the voting age population, while controlling for whether a person voted in the prior midterm primary. This is achieved using panel data that captures voting histories, reflecting the habitual nature of voting [28]. We incorporate lagged voting to consider how past values of the outcome variable influence its present value, as seen in voting behavior. These recursive models with panel data are robust as they model the change in the probability of turnout at the individual level [29]. Consequently, the outcome variable represents the estimated difference in the change in participation rates between individuals in states with nonpartisan primaries versus closed primaries.

The main explanatory variable is whether the respondent resides in a state that has adopted and implemented a nonpartisan primary (top-two or top-four), fully open primary, or partially open primary, defined as open for unaffiliated voters, using data from the 2021 National Conference of State Legislatures (NCSL, Primary Types Table). States that have adopted primary reform are coded as 1, while all other jurisdictions are coded as 0. States with closed or semi-closed primaries serve as the reference category [1].

### Demographic subgroup models

We use these data to measure the share of the electorate broken into three groups-- young (18–34), middle-aged (35–64), and older voters (65 years plus)—that voted in the 2022 primary elections. These age thresholds were the result of the terciles of the population. Alternative age threshold results in similar findings. Income from the credit bureau reports is broken into four quartiles (below $30K annual income, $30-75K, $75-100K, and $100K plus) to measure shares of the population from low- and high-income groups. These are common breaks for reporting demographic data used by Pew and other organizations. Education is measured in terciles by low, medium and higher probability of having a college degree.

### Controlling for electoral competition

A significant problem with primary elections, beyond the very low turnout and wide variation in primary rules, is that competition varies dramatically from state to state and over time [4,30]. A key factor in studying participation in primary electorates is how competitive the contests are and the funding available to mobilize voters to turnout [8,31,32]. Considering this, we control how competitive the Senate or gubernatorial races were in the respondent’s state. Competitive Senate or governor primaries are measured by fractionalization; the intuition is that these two types of *statewide* elections are likely to drive turnout, while other statewide elections (Lieutenant Governor, Attorney General, etc.) or elections in particular districts are less likely to affect turnout. Fractionalization is measured using a 0–1 index developed by Canon [33] with data as reported by Micatka et al. [15] for each state for both the Democratic and Republican parties in the 2022 primaries. Larger index scores correspond to competitive races with more than two candidates; the larger the number of similarly competitive candidates, the closer the index is to 1. This index is operationalized as F = 1 - ∑ [(C_1_)^2^ + (C_2_)^2^+ (C_3_)^2^+ (C_4_)^2^...] where F is the fractionalization index, C_1_ is the percentage of the total vote received by the first candidate, C_2_ is the percentage of the total vote received by the second candidate, and so on. A one-candidate race has a fractionalization index of zero, a race where two candidates split the vote would have a fractionalization index of 0.5 (or 1 – (0.5^2^ + 0.5^2^). States with no contests have no electoral competition. The Supplemental Information, Tables A4-A6 (S2 File), demonstrate that our main substantive results are unchanged when the control for electoral competition is omitted.

In terms of other factors that could predict voting in primaries, we control for gender included in the voter files measured by females coded 1 and males 0. These data also include modeled estimates of an individual’s race (in some states race is reported on the state voter files). The statistical models include binary variables for Black, Latino, and other race (coded 1, all others 0), marital status (married coded 1, non-married 0). The administrative credit bureau records are more accurate than self-reported income. The models include the probability of having a college degree using administrative data. The analysis includes probabilistic political ideology with a high probability of being a strong liberal; this variable is provided to researchers by Catalist and is based on national survey data, canvassing data, campaign contributions, commercial sources, etc. Previous studies have shown these demographic data are reliable [23–24], when validated and matched with national survey data [6]. The models control for restrictive state voting laws in the state measured by the 2020 Cost of Voting Index (COVI, costofvotingindex.com) [34].

### Results by age groups

We used three logistic regression models estimated with the panel data to predict whether an individual voted in the 2022 primaries, controlling for whether the individual voted in the 2018 primaries with a lagged variable. Supplemental Information Table A1 (S2 File) presents results from these models for the three age group subsamples: young people, middle-aged, and older. By including a variable that indicates whether the individual voted in the previous midterm-only primary, the models assess the change in voter turnout at the individual level, yielding more causal insights. The coefficients are reported as unstandardized logistic regression coefficients, with robust standard errors in parentheses.

Column 1 reports turnout of young people, column 2 shows results of models turnout of middle-aged voters, and column 3 applies to adults aged 65 and above (See Supplemental Information Table A1 (S2 File)). As expected, those who voted in the 2018 primary are more likely to participate in the 2022 primaries across age groups. Among the young and middle-aged subsamples, women were more likely to vote in primaries than men. Nationally, white individuals are generally more likely to vote in primaries than people of color. Holding these factors plus electoral competition constant, individuals living in states with open primaries are not statistically different compared to those in the reference group (closed or semi-closed primary states). Conversely, residents of the four Midwest states with partially open primaries were less likely to vote in the 2022 primaries than people in closed primaries.

Table A1 (S2 File) illustrates that, across the age cohorts, individuals living in states with nonpartisan (or all-candidate) primaries—where Republicans and Democrats appear on a single ballot—were significantly more likely to vote, other things held constant. This effect is observed for the subsample of young, middle-aged, and older voters. Living in a state with open primaries or partially open primaries was not associated with higher turnout among any category of education.

What are the substantive effects of state primary type on voting in the 2022 primaries? As reported in Table 1, the predicted probability of voting in the 2022 primary is 8.5 percentage points higher for young people aged 18–34 in nonpartisan states than in closed primary states (see Table 1). Put differently, turnout among the youngest cohort was 2.4x greater (i.e.,.144/ 0.59 = 2.44) in nonpartisan primaries than closed primaries. While the average turnout nationally was very low for this group (9%), the percentage point increase in turnout in nonpartisan primaries relative to the group’s baseline turnout was 94% (i.e., 8.5/ 9.02). for the youngest voters. People 35–64 living in nonpartisan primary states were 13.4 percentage points more likely to vote, or 1.97x greater than the national average for this group. Primary electorates already heavily skew towards older people, and their turnout is also higher in nonpartisan primaries. The oldest cohort was 13.2 percentage points more likely to vote when living in states with a nonpartisan primary, or 1.57x greater than the national average turnout for this cohort. These results are generally consistent with those reported in a case study of changes in Alaska’s primary electorate before and after adoption of the nonpartisan primary [6]. Young people appear to especially benefit from nonpartisan primaries.

**Table 1:** Predicted probability of voting in the 2022 primary for age groups, all else equal, including vote history.

	Age 18–34	Age 35–64	Age 65+
Prob. voting closed primary	.059 (.005)	.138 (.011)	.231 (.018)
Prob. voting nonpartisan primary	.144 (.134)	.272 (.019)	.363 (.023)
Percentage point difference in prob. of turnout	8.5	13.4	13.2
Mean turnout rate in 2022 primary	9.02%	19.28%	29.87%
% pt diff in voting/group’s baseline turnout	94%	69%	44%
Ratio of turnout nonpartisan vs. closed primary	2.44	1.97	1.57

Note: Estimated from models Table A1 in Supplemental Information (S2 File); robust standard errors of predictions in parentheses.

### Results by income groups

While age biases in primary electorates can lead to representation bias, just as critical may be income biases in primary electorates that tend to skew toward the affluent. Supplemental Information Table A2 (S2 File) repeats the same set of models but instead estimates logistic regression models predicting 2022 primary voting for subsamples of individuals defined by quartiles of income, from low to high. Despite a turnout rate of only 9% nationally among the lowest-income voters ($30K and below), the results show that low-income people living in nonpartisan primary states were significantly more likely to vote. Open primaries were not linked to higher turnout for people in different income groups. Across the board, residents of states with nonpartisan primaries were more likely to vote in primaries than those in closed primary states.

Table 2 shows the predicted effect of turnout by income category, holding other factors constant. As with age, turnout in nonpartisan primaries is higher among all categories of income. Among people earning $30K and less, those living in states with the nonpartisan primary were 4.7 percentage points more likely to vote, or a 52% increased probability of participating over the group’s relatively low average turnout nationally. For people in the middle- and highest-income groups, living in a nonpartisan primary state is associated with significant increases in turnout. Notably, even people earning between $30 and 70K annually have a 13-percentage point increased probability of voting in the primary, a substantively large effect.

**Table 2:** Predicted probability of voting in the 2022 primary for income groups, all else equal, including vote history.

	Lowest Income Under $30K	Second Lowest $30K-$75K	Third Lowest $75K-$100K	Highest $100K plus
Prob. voting closed primary	.049 (.004)	.140 (.011)	.240 (.021)	.274 (.027)
Prob. voting nonpartisan primary	.096 (.008)	.271 (.017)	.444 (.026)	.521 (.028)
Percentage point difference in prob. of turnout	4.7	13.1	20.4	24.7
Mean turnout rate in 2022 primary	8.99%	18.95%	29.02%	33.25%
% pt diff in voting/group’s baseline turnout	52%	69%	70%	74%
Ratio of turnout nonpartisan vs. closed primary	1.95	1.93	1.85	1.90

Note: Estimated from models in Supplemental Information Table A2 (S2 File); robust standard errors of predictions in parentheses.

### Results by education groups

Finally, Table A3 in the Supplemental Information (S2 File) shows the results for subsamples of the US adult population broken down by the probability of having a college degree (low, medium, and high), with the same set of control variables for individual-level factors, primary competition, prior vote history, etc. Contrary to expectations, the open primary again is not related to a higher probability of voting in the 2022 primary for any education group, but living in a state with a nonpartisan primary is. These results hold varying model specifications. For all education groups (low, medium and high probability of a college degree), the nonpartisan primary was associated with a substantively meaningful boost in the probability of primary turnout, ranging 9–24 percentage points, over living in a state with a close primary (reference group). However, the nonpartisan primary appears to have the largest substantive effect in increasing the turnout of college-educated voters (see Table 3).

**Table 3:** Predicted probability of voting in the 2022 primary for education groups (prob college degree or higher), all else equal, including vote history.

	Low educ.	Medium educ.	High educ.
Prob. voting closed primary	.115 (.009)	.178 (.017)	.209 (.020)
Prob. voting nonpartisan primary	.207 (.015)	.368 (.024)	.451 (.029)
Percentage point difference in prob. of turnout	9.2	19	24.2
Mean turnout rate in 2022 primary	16.65%	24.14%	28.18%
% pt diff in voting/ group’s baseline turnout	55%	79%	86%
Ratio of turnout nonpartisan vs. closed primary	1.80	2.07	2.16

Note: Estimated from models in Supplemental Information Table A3 (S2 File); robust standard errors of predictions in parentheses.

## Discussion and conclusion

Recent studies of turnout in primaries have found that while primary turnout is generally low, it may be higher in states with nonpartisan primaries [16], and particularly higher in nonpartisan primary states than in closed primary states [15]. General election turnout [35] is much higher than primary election turnout. Further, the gap between primary and general election turnout has been found to be greater in closed primaries than non-partisan primaries [14].

We add to the emerging understanding of turnout across various primary types by analyzing what we suggest are the best available data for examining differences in turnout across demographic groups across nonpartisan, open, and closed primary systems. Across age, education, and income groups, with family income measured by credit bureau reports, we find that turnout is higher in nonpartisan primaries than in closed primaries. When open primary states are compared to closed primary states, we find no differences in turnout across these demographic groups, apart from higher turnout for people in the lowest income category.

A key question motivating this study was if open and/or nonpartisan primaries were associated with differentially higher turnout across demographic groups who may be underrepresented in elections, particularly primary elections. We do find an asymmetric relationship between primary type and primary turnout by age cohort. There was a higher likelihood of turnout for all age categories under nonpartisan primaries than under closed primaries; however, we observed the greatest proportionate difference between turnout in nonpartisan primaries versus the national average among younger voters, and the least among older voters. Nonpartisan primaries had proportionately even relationships with higher turnout across income groups, but since the affluent are more likely to vote in primaries, this may benefit lower-income groups. Additional studies of these relationships could benefit from examining future election years after New Mexico and other states implement their primary system changes in 2026.

US primary elections are notable for socioeconomic gaps in participation rates [8]. Consequences of this political inequality include the distortion of public policy where the preferences of certain groups are amplified by closed primary elections [36], and the resulting policies and candidates reflect the interests of the more powerful groups. This can lead to policies that benefit various categories of partisans over people with weaker party affiliations, but also the older and wealthier at the expense of the less privileged [37,38]. Another consequence of political inequality is the erosion of trust in government. When citizens feel that their voices are not being heard, they are less likely to trust their elected officials and the political process as a whole. This can lead to increased cynicism, apathy, and ultimately, a decline in democratic participation [38].

Political scientists and policymakers don’t often connect primary election rules as contributing to political inequality, but this study suggests that certain types of state primary rules, including nonpartisan primaries, are associated with lower political inequalities in the age of who votes, but that various primary rules largely do not eliminate inequality in the representativeness of primary electorates. To our knowledge, this is one of the first studies to use voter file data to measure individual-level primary turnout for different demographic groups relative to state primary rules.

## Supporting information

S1 File PLOS-One Stata do replication code. (PDF)

S2 File Supplemental information (Appendix Tables A1–A9). (PDF)
