# Supplementary material for: Can nonpartisan primaries boost turnout and lessen demographic disparities?
Source: PLoS One. 2025 Dec 8;20(12):e0335840. doi: 10.1371/journal.pone.0335840 (PMC12685176; doi:10.1371/journal.pone.0335840)
Supplement: S1 File — (PDF) [file pone.0335840.s001.pdf]

```
 *****************
 *** 2023 File ***
 *****************

**** VARIABLE ****
** reading in file

** dropping deceased observations
drop if deadwood==1

/* creating variables */
** Creating Proper Race Variables
tab race, gen(race_categ)
rename race_categ1 asian_fix
rename race_categ2 black_nh_fix
rename race_categ3 white_nh_fix
rename race_categ4 hisp_fix
rename race_categ6 race_other_nh_fix
replace  race_other_nh_fix=1 if race_categ5==1
replace race_other_nh_fix=1 if race_categ7==1

tab asian_fix
tab black_nh_fix
tab white_nh_fix
tab hisp_fix
tab race_other_nh_fix

*** Bounding Age
tab age_bounded_100
replace age_bounded_100=. if age==.
tab age_bounded_100

tab age_bounded_100_sq
replace age_bounded_100_sq=. if age==.
tab age_bounded_100_sq

tab age_categ
replace age_categ=. if age==.
tab age_categ

** Merging in State Primary Type Data

* Dropping VA and LA data
drop if state_identification==51
drop if state_identification==22

** Creating Open Unaffl/Partially Open
gen part_open_primary_2022=0
        replace part_open_primary_2022=1 if state_identification==17
        replace part_open_primary_2022=1 if state_identification==18
        replace part_open_primary_2022=1 if state_identification==19
        replace part_open_primary_2022=1 if state_identification==39
 tab part_open_primary_2022
        replace part_open_primary_2022=. if open_primary_2022==.
 tab part_open_primary_2022

 gen open_primary_2022_v2=open_primary_2022
        replace open_primary_2022_v2=0 if state_identification==17
        replace open_primary_2022_v2=0 if state_identification==18
        replace open_primary_2022_v2=0 if state_identification==19
        replace open_primary_2022_v2=0 if state_identification==39


 *** Merging in State Frac Data
 drop _merge
 merge m:1 state_identification using state_frac_data.dta

 ** Merging in COVI and EPI
 drop _merge
```

```
* keeping only variables we need for models so they run faster
keep age_bounded_100  age_bounded_100_sq female white_nh_fix asian_fix black_nh_fix hisp_fix
race_other_nh_fix married educ_bach_degree income liberal conservative pid3_catalist deadwood
state_identification closed_primary_2014 closed_primary_2016 closed_primary_2018
closed_primary_2020 closed_primary_2022 open_primary_2014 open_primary_2016 open_primary_2018
open_primary_2020 open_primary_2022 np_primary_2014 np_primary_2016 np_primary_2018
np_primary_2020 np_primary_2022 dgfrac_2014 rgfrac_2014 dsfrac_2014 rsfrac_2014 dsfrac_2018
rsfrac_2018 dgfrac_2018 rgfrac_2018 dgfrac_2022 rgfrac_2022 dsfrac_2022 rsfrac_2022
catalistmodel_partisanship catalistmodel_biden_support catalistmodel_ideology_plus e2022pep
e2018pep vote_2022p vote_2018p educ_prob_college FinalCOVI2022 InitialCOVI2022 FinalCOVI2020
InitialCOVI2020 FinalCOVI2016 InitialCOVI2016 open_primary_2022 part_open_primary_2022
open_primary_2022_v2


** Creating Age LMH Variable
gen age_lmh=.
	replace age_lmh=1 if age_bounded_100>17 & age_bounded_100<35
	replace age_lmh=2 if age_bounded_100>34 & age_bounded_100<65
	replace age_lmh=3 if age_bounded_100>64
tab age_lmh

label define agelmh_labels 1 "18-34" 2 "35-64" 3 "65+"

label values age_lmh agelmh_labels
tab age_lmh

** Creating Income LMH Variable
gen income_lmh=.
	replace income_lmh=1 if income==0
	replace income_lmh=1 if income==1
	replace income_lmh=2 if income==2
	replace income_lmh=2 if income==3
	replace income_lmh=3 if income==4
	replace income_lmh=4 if income==5
	replace income_lmh=4 if income==6
tab income_lmh

label define incomelmh_labels 1 "Less than $30,000" 2 "$30,000 - $75,000" 3 "$75,000 - $100,000"
4 "$100,000+"

label values income_lmh incomelmh_labels
tab income_lmh

** Creating Education LMH Variable
gen educ_lmh=.
	replace educ_lmh=1 if educ_prob_college<34
	replace educ_lmh=2 if educ_prob_college>33 & educ_prob_college<67
	replace educ_lmh=3 if educ_prob_college>66
tab educ_lmh

** Standardize COVI
gen rescaled_covi2020 = (FinalCOVI2020 - -2.916981) / (1.436939 - -2.916981)
sum rescaled_covi2020

** Creating Single Fract. Variable
alpha rsfrac_2022 rgfrac_2022 dsfrac_2022  dgfrac_2022, std gen(fract_scale) det
sum fract_scale


***************************
**** DESCRIPTIVES ****
***************************
** 2022 Primary - Closed States
tab vote_2022p if closed_primary_2022== 1 /* Turnout */

sum catalistmodel_partisanship if closed_primary_2022==1 & vote_2022p==1
sum catalistmodel_ideology_plus if closed_primary_2022==1 & vote_2022p==1

* 2022 Primary Demographics - Closed States
tab vote_2022p age_categ if closed_primary_2022==1, col
```

```
tab vote_2022p educ_bach_degree if closed_primary_2022==1, col

tab vote_2022p female if closed_primary_2022==1, col


tab vote_2022p married if closed_primary_2022==1, col

tab vote_2022p income if closed_primary_2022==1, col

tab vote_2022p white_nh_fix if closed_primary_2022==1 & white_nh_fix==1, col
tab vote_2022p black_nh_fix if closed_primary_2022==1 & black_nh_fix==1, col
tab vote_2022p hisp_fix if closed_primary_2022==1 & hisp_fix==1, col
tab vote_2022p asian_fix if closed_primary_2022==1 & asian_fix==1, col
tab vote_2022p race_other_nh_fix if closed_primary_2022==1 & race_other_nh_fix==1, col

tab vote_2022p if closed_primary_2022==1 & pid3_catalist==1 /* republican */
tab vote_2022p if closed_primary_2022==1 & pid3_catalist==2 /* independent */
tab vote_2022p if closed_primary_2022==1 & pid3_catalist==3 /* democrat */


tab vote_2022p conservative if closed_primary_2022==1 & conservative==1, col /* conserv */
tab vote_2022p moderate if closed_primary_2022==1 & moderate==1, col /* mod */
tab vote_2022p liberal if closed_primary_2022==1 & liberal==1, col /* lib */

** 2022 Primary Overall - Open States
tab vote_2022p if open_primary_2022== 1 /* Turnout */

sum catalistmodel_partisanship if open_primary_2022==1 & vote_2022p==1
sum catalistmodel_ideology_plus if open_primary_2022==1 & vote_2022p==1

* 2022 Primary Demographics - Open States
tab vote_2022p age_categ if open_primary_2022==1, col

tab vote_2022p educ_bach_degree if open_primary_2022==1, col

tab vote_2022p female if open_primary_2022==1, col


tab vote_2022p married if open_primary_2022==1, col

tab vote_2022p income if open_primary_2022==1, col

tab vote_2022p white_nh_fix if open_primary_2022==1 & white_nh_fix==1, col
tab vote_2022p black_nh_fix if open_primary_2022==1 & black_nh_fix==1, col
tab vote_2022p hisp_fix if open_primary_2022==1 & hisp_fix==1, col
tab vote_2022p asian_fix if open_primary_2022==1 & asian_fix==1, col
tab vote_2022p race_other_nh_fix if open_primary_2022==1 & race_other_nh_fix==1, col

tab vote_2022p if open_primary_2022==1 & pid3_catalist==1 /* republican */
tab vote_2022p if open_primary_2022==1 & pid3_catalist==2 /* independent */
tab vote_2022p if open_primary_2022==1 & pid3_catalist==3 /* democrat */


tab vote_2022p conservative if open_primary_2022==1 & conservative==1, col /* conserv */
tab vote_2022p moderate if open_primary_2022==1 & moderate==1, col /* mod */
tab vote_2022p liberal if open_primary_2022==1 & liberal==1, col /* lib */


** 2022 Primary Overall - Nonpartisan primary States
tab vote_2022p if np_primary_2022== 1 /* Turnout */

sum catalistmodel_partisanship if np_primary_2022==1 & vote_2022p==1
sum catalistmodel_ideology_plus if np_primary_2022==1 & vote_2022p==1

* 2022 Primary Demographics - Nonpartisan primary States
tab vote_2022p age_categ if np_primary_2022==1, col

tab vote_2022p educ_bach_degree if np_primary_2022==1, col

tab vote_2022p female if np_primary_2022==1, col
```

```
tab vote_2022p married if np_primary_2022==1, col

tab vote_2022p income if np_primary_2022==1, col

tab vote_2022p white_nh_fix if np_primary_2022==1 & white_nh_fix==1, col
tab vote_2022p black_nh_fix if np_primary_2022==1 & black_nh_fix==1, col
tab vote_2022p hisp_fix if np_primary_2022==1 & hisp_fix==1, col
tab vote_2022p asian_fix if np_primary_2022==1 & asian_fix==1, col
tab vote_2022p race_other_nh_fix if np_primary_2022==1 & race_other_nh_fix==1, col

tab vote_2022p if np_primary_2022==1 & pid3_catalist==1 /* republican */
tab vote_2022p if np_primary_2022==1 & pid3_catalist==2 /* independent */
tab vote_2022p if np_primary_2022==1 & pid3_catalist==3 /* democrat */


tab vote_2022p conservative if np_primary_2022==1 & conservative==1, col /* conserv */
tab vote_2022p moderate if np_primary_2022==1 & moderate==1, col /* mod */
tab vote_2022p liberal if np_primary_2022==1 & liberal==1, col /* lib */


************************************
*** Turnout By Demographic Group ***
************************************
tab vote_2022p if age_lmh==1
tab vote_2022p if age_lmh==2
tab vote_2022p if age_lmh==3

tab vote_2022p if income_lmh==1
tab vote_2022p if income_lmh==2
tab vote_2022p if income_lmh==3
tab vote_2022p if income_lmh==4

tab vote_2022p if educ_lmh==1
tab vote_2022p if educ_lmh==2
tab vote_2022p if educ_lmh==3


*******************
*** MAIN MODELS ***
*******************
**** MODELS FOR AGE, INCOME, AND EDUCATION ARE BELOW
**** AGE MODELS
* 18-34 Subsample
logit vote_2022p vote_2018p i.np_primary_2022 i.open_primary_2022_v2 i. part_open_primary_2022
female black_nh_fix asian_fix hisp_fix race_other_nh_fix income educ_prob_college married
catalistmodel_ideology_plus rsfrac_2022 rgfrac_2022 dsfrac_2022  dgfrac_2022  FinalCOVI2020 if
age_lmh==1, cluster(state_identification)
estimates store model_age_low

* 35-64 Subsample
logit vote_2022p vote_2018p i.np_primary_2022 i.open_primary_2022_v2 i. part_open_primary_2022
female black_nh_fix asian_fix hisp_fix race_other_nh_fix income educ_prob_college married
catalistmodel_ideology_plus rsfrac_2022 rgfrac_2022 dsfrac_2022  dgfrac_2022 FinalCOVI2020 if
age_lmh==2, cluster(state_identification)
estimates store model_age_mid

* 65+ Subsample
logit vote_2022p vote_2018p i.np_primary_2022 i.open_primary_2022_v2 i. part_open_primary_2022
female black_nh_fix asian_fix hisp_fix race_other_nh_fix income educ_prob_college married
catalistmodel_ideology_plus rsfrac_2022 rgfrac_2022 dsfrac_2022  dgfrac_2022 FinalCOVI2020 if
age_lmh==3, cluster(state_identification)
estimates store model_age_high

**** INCOME MODELS
* Less than 30,000 Subsample
logit vote_2022p vote_2018p i.np_primary_2022 i.open_primary_2022_v2 i. part_open_primary_2022
age_bounded_100 age_bounded_100_sq female black_nh_fix asian_fix hisp_fix race_other_nh_fix
educ_prob_college married catalistmodel_ideology_plus rsfrac_2022 rgfrac_2022 dsfrac_2022
dgfrac_2022 FinalCOVI2020 if income_lmh==1, cluster(state_identification)
estimates store model_income_low
```

```
* 30,000-75,000 Subsample
logit vote_2022p vote_2018p i.np_primary_2022 i.open_primary_2022_v2 i. part_open_primary_2022
age_bounded_100 age_bounded_100_sq female black_nh_fix asian_fix hisp_fix race_other_nh_fix
educ_prob_college married catalistmodel_ideology_plus rsfrac_2022 rgfrac_2022 dsfrac_2022
dgfrac_2022 FinalCOVI2020 if income_lmh==2, cluster(state_identification)
estimates store model_income_mid

* 75,000 - 100,000 Subsample
logit vote_2022p vote_2018p i.np_primary_2022 i.open_primary_2022_v2 i. part_open_primary_2022
age_bounded_100 age_bounded_100_sq female black_nh_fix asian_fix hisp_fix race_other_nh_fix
educ_prob_college married catalistmodel_ideology_plus rsfrac_2022 rgfrac_2022 dsfrac_2022
dgfrac_2022 FinalCOVI2020 if income_lmh==3, cluster(state_identification)
estimates store model_income_high

* 100,000+ Subsample
logit vote_2022p vote_2018p i.np_primary_2022 i.open_primary_2022_v2 i. part_open_primary_2022
age_bounded_100 age_bounded_100_sq female black_nh_fix asian_fix hisp_fix race_other_nh_fix
educ_prob_college married catalistmodel_ideology_plus rsfrac_2022 rgfrac_2022 dsfrac_2022
dgfrac_2022 FinalCOVI2020 if income_lmh==4, cluster(state_identification)
estimates store model_income_highest

**** EDUCATION MODELS
* Lower Third Subsample
logit vote_2022p vote_2018p i.np_primary_2022 i.open_primary_2022_v2 i. part_open_primary_2022
age_bounded_100 age_bounded_100_sq female black_nh_fix asian_fix hisp_fix race_other_nh_fix
income  married catalistmodel_ideology_plus rsfrac_2022 rgfrac_2022 dsfrac_2022  dgfrac_2022
FinalCOVI2020 if educ_lmh==1, cluster(state_identification)
estimates store model_educ_low

* Middle Third Subsample Subsample
logit vote_2022p vote_2018p i.np_primary_2022 i.open_primary_2022_v2 i. part_open_primary_2022
age_bounded_100 age_bounded_100_sq female black_nh_fix asian_fix hisp_fix race_other_nh_fix
income  married catalistmodel_ideology_plus rsfrac_2022 rgfrac_2022 dsfrac_2022  dgfrac_2022
FinalCOVI2020 if educ_lmh==2, cluster(state_identification)
estimates store model_educ_mid

* Highest Third Subsample
logit vote_2022p vote_2018p i.np_primary_2022 i.open_primary_2022_v2 i. part_open_primary_2022
age_bounded_100 age_bounded_100_sq female black_nh_fix asian_fix hisp_fix race_other_nh_fix
income  married catalistmodel_ideology_plus rsfrac_2022 rgfrac_2022 dsfrac_2022  dgfrac_2022
FinalCOVI2020 if educ_lmh==3, cluster(state_identification)
estimates store model_educ_high


*******************************
*** Predicted Probabilities ***
*******************************
* Low Age Group
estimates restore model_age_low
margins, at(np_primary_2022==0 open_primary_2022==0 part_open_primary_2022==0) atmeans
margins, at(np_primary_2022==1 open_primary_2022==0 part_open_primary_2022==0) atmeans

* Middle Age Group
estimates restore model_age_mid
margins, at(np_primary_2022==0 open_primary_2022==0 part_open_primary_2022==0) atmeans
margins, at(np_primary_2022==1 open_primary_2022==0 part_open_primary_2022==0) atmeans

* Highest Age Group
estimates restore model_age_high
margins, at(np_primary_2022==0 open_primary_2022==0 part_open_primary_2022==0) atmeans
margins, at(np_primary_2022==1 open_primary_2022==0 part_open_primary_2022==0) atmeans

* Low Income Group
estimates restore model_income_low
margins, at(np_primary_2022==0 open_primary_2022==0 part_open_primary_2022==0) atmeans
margins, at(np_primary_2022==1 open_primary_2022==0 part_open_primary_2022==0) atmeans

* Middle Income Group
estimates restore model_income_mid
margins, at(np_primary_2022==0 open_primary_2022==0 part_open_primary_2022==0) atmeans
```

```
margins, at(np_primary_2022==1 open_primary_2022==0 part_open_primary_2022==0) atmeans

* High Income Group
estimates restore model_income_high
margins, at(np_primary_2022==0 open_primary_2022==0 part_open_primary_2022==0) atmeans
margins, at(np_primary_2022==1 open_primary_2022==0 part_open_primary_2022==0) atmeans

* Highest Income Group
estimates restore model_income_highest
margins, at(np_primary_2022==0 open_primary_2022==0 part_open_primary_2022==0) atmeans
margins, at(np_primary_2022==1 open_primary_2022==0 part_open_primary_2022==0) atmeans

* Low Educ Group
estimates restore model_educ_low
margins, at(np_primary_2022==0 open_primary_2022==0 part_open_primary_2022==0) atmeans
margins, at(np_primary_2022==1 open_primary_2022==0 part_open_primary_2022==0) atmeans

* Middle Educ Group
estimates restore model_educ_mid
margins, at(np_primary_2022==0 open_primary_2022==0 part_open_primary_2022==0) atmeans
margins, at(np_primary_2022==1 open_primary_2022==0 part_open_primary_2022==0) atmeans

* High Educ Group
estimates restore model_educ_high
margins, at(np_primary_2022==0 open_primary_2022==0 part_open_primary_2022==0) atmeans
margins, at(np_primary_2022==1 open_primary_2022==0 part_open_primary_2022==0) atmeans


*************************
*** Regression Tables ***
*************************
** AGE TABLE
esttab model_age_low  model_age_mid  model_age_high using AgeCateg_NPP_Table_2023File.csv,
replace se(3) label  ///
               b(%9.2f) star(* .1 ** .05 *** .01) nolz nobase nogaps s(N ll r2_p bic) ///
               varlabels(female "Female" asian_fix "Asian" black_nh_fix "Black" hisp_fix "Latino"
///
               race_other_nh_fix "Race Other" married "Married" educ_prob_college "Pr(Bachelor's
degree)" income "Income" catalistmodel_ideology_plus ///
               "Catalist Ideology" _cons "Constant" 1.part_open_primary_2022 "Partially Open
Primary State" 1.open_primary_2022_v2 "Open Primary State" ///
               1.np_primary_2022 "Non-partisan Primary State" ///
               rsfrac_2022 "Rep. Senate Fract. 2022" dsfrac_2022 "Dem. Senate Fract. 2022" ///
               rgfrac_2022 "Rep. Governor Fract. 2022" dgfrac_2022 "Dem. Governor Fract. 2022"
vote_2018p "Vote in 2018 Primary" FinalCOVI2020 "COVI 2020") ///
               mtitles("Overall" "Republicans" ///
               "Independents" "Democrats" ) ///
               order(vote_2018p 1.np_primary_2022 1.open_primary_2022_v2 1.part_open_primary_2022
female asian_fix black_nh_fix hisp_fix race_other_nh_fix ///
               married educ_prob_college income catalistmodel_ideology_plus dsfrac_2022
dgfrac_2022 rsfrac_2022 rgfrac_2022 FinalCOVI2020)

** INCOME TABLE
esttab model_income_low  model_income_mid  model_income_high model_income_highest using
IncomeCateg_NPP_Table_2023File.csv, replace se(3) label  ///
               b(%9.2f) star(* .1 ** .05 *** .01) nolz nobase nogaps s(N ll r2_p bic) ///
               varlabels(age_bounded_100 "Age" age_bounded_100_sq "Age Squared" female "Female"
asian_fix "Asian" black_nh_fix "Black" hisp_fix "Latino" ///
               race_other_nh_fix "Race Other" married "Married" educ_prob_college "Pr(Bachelor's
degree)" catalistmodel_ideology_plus ///
               "Catalist Ideology" _cons "Constant" 1.part_open_primary_2022 "Partially Open
Primary State" 1.open_primary_2022_v2 "Open Primary State" ///
               1.np_primary_2022 "Non-partisan Primary State" ///
               rsfrac_2022 "Rep. Senate Fract. 2022" dsfrac_2022 "Dem. Senate Fract. 2022" ///
               rgfrac_2022 "Rep. Governor Fract. 2022" dgfrac_2022 "Dem. Governor Fract. 2022"
vote_2018p "Vote in 2018 Primary" FinalCOVI2020 "COVI 2020") ///
               mtitles("Low" "Mid" "High" "Highest" ) ///
               order(vote_2018p 1.np_primary_2022 1.open_primary_2022_v2 1.part_open_primary_2022
age_bounded_100 age_bounded_100_sq female asian_fix ///
               black_nh_fix hisp_fix race_other_nh_fix married educ_prob_college
catalistmodel_ideology_plus dsfrac_2022 dgfrac_2022 rsfrac_2022 rgfrac_2022 FinalCOVI2020)
```

```
** EDUC TABLE
esttab model_educ_low  model_educ_mid  model_educ_high using EducCateg_NPP_Table_2023File.csv,
replace se(3) label  ///
              b(%9.2f) star(* .1 ** .05 *** .01) nolz nobase nogaps s(N ll r2_p bic) ///
              varlabels(age_bounded_100 "Age" age_bounded_100_sq "Age Squared" female "Female"
asian_fix "Asian" black_nh_fix "Black" hisp_fix "Latino" ///
              race_other_nh_fix "Race Other" married "Married" income "Income"
catalistmodel_ideology_plus ///
              "Catalist Ideology" _cons "Constant" 1.part_open_primary_2022 "Partially Open
Primary State" 1.open_primary_2022_v2 "Open Primary State" ///
              1.np_primary_2022 "Non-partisan Primary State" ///
              rsfrac_2022 "Rep. Senate Fract. 2022" dsfrac_2022 "Dem. Senate Fract. 2022" ///
              rgfrac_2022 "Rep. Governor Fract. 2022" dgfrac_2022 "Dem. Governor Fract. 2022"
vote_2018p "Vote in 2018 Primary" FinalCOVI2020 "COVI 2020") ///
              mtitles("Low" "Mid" "High" "Highest" ) ///
              order(vote_2018p 1.np_primary_2022 1.open_primary_2022_v2 1.part_open_primary_2022
age_bounded_100 age_bounded_100_sq female asian_fix ///
              black_nh_fix hisp_fix race_other_nh_fix married income catalistmodel_ideology_plus
dsfrac_2022 dgfrac_2022 rsfrac_2022 rgfrac_2022 FinalCOVI2020)
```
