# Supplementary material for: Can nonpartisan primaries boost turnout and lessen demographic disparities?
Source: PLoS One. 2025 Dec 8;20(12):e0335840. doi: 10.1371/journal.pone.0335840 (PMC12685176; doi:10.1371/journal.pone.0335840)
Supplement: S2 File — (PDF) [file pone.0335840.s002.pdf]

Supplemental Information

Table of Contents

1. Appendix Table A1
2. Appendix Table A2
3. Appendix Table A3
4. Appendix Table A4
5. Appendix Table A5
6. Appendix Table A6
7. Appendix Table A7
8. Introduction: Merging Voter File and Survey Data
9. Appendix Table A8
10. Appendix Table A9

Appendix Table A1: Probability of voting in the 2022 primaries, lagging turnout in the 2018 primaries, nationwide and for age groups 1% sample voter file data

	(1)	(2)	(3)
	**Ages 18- 34**	**Ages 35-64**	**Ages 65+**
Vote in 2018 Primary	1.95***	2.21***	2.71***
	(.058)	(.034)	(.050)
Non-partisan Primary State	.98***	.85***	.64***
	(.153)	(.160)	(.166)
Open Primary State	.13	.08	.08
	(.146)	(.127)	(.122)
Partially Open Primary State	-.35**	-.43***	-.37***
	(.161)	(.094)	(.100)
Female	.07***	.05***	.02
	(.022)	(.013)	(.012)
Asian	-.50***	-.71***	-.89***
	(.053)	(.076)	(.152)
Black	-.71***	-.29***	-.27***
	(.039)	(.061)	(.082)
Latino	-.70***	-.67***	-.85***
	(.070)	(.047)	(.060)
Race Other	-.40***	-.38***	-.55***
	(.037)	(.044)	(.069)
Married	.03	.06***	.15***
	(.031)	(.019)	(.023)
Pr(Bachelor's degree)	-.001	-.0004	.003**
	(.001)	(.001)	(.001)
Income	.35***	.27***	.19***
	(.026)	(.022)	(.020)
Political Ideology	.01***	.004**	.01***
	(.002)	(.002)	(.002)
Dem. Senate Fract. 2022	.69	.47	.56**
	(.547)	(.340)	(.274)
Dem. Governor Fract. 2022	-.14	-.12	-.26
	(.194)	(.175)	(.193)
Rep. Senate Fract. 2022	.24	.34	.19
	(.250)	(.230)	(.229)
Rep. Governor Fract. 2022	.33*	.36*	.49**
	(.190)	(.204)	(.215)
COVI 2020	-.01	.04	-.03

	(.066)	(.058)	(.052)
Constant	-4.27***	-3.31***	-2.99***
	(.193)	(.128)	(.125)
Observations	582,988	1,164,623	765,798
Log-likelihood	-154958.89	-458526.84	-331544.89
Pseudo R2	.12	.20	.29
BIC	310170.02	917319.06	663347.20

Catalist voter file data 2023. Unstandardized logistic regression coefficients. Standard errors are clustered by state in parentheses. * $p < .01$, ** $p < .05$, *** $p < .001$

Appendix Table A2: Probability of voting in the 2022 primaries, lagging turnout in the 2018 primaries, nationwide and for income quartiles, 1% sample voter file data

	(1)	(2)	(3)	(4)
	**Income Less than $30,000**	**Income Between $30,000 and $75,000**	**Income Between $75,000 and $100,000**	**Income Above $100,000**
Vote in 2018 Primary	2.44***	2.27***	2.12***	1.98***
	(.042)	(.030)	(.035)	(.035)
Non-partisan Primary State	.73***	.83***	.93***	1.06***
	(.138)	(.153)	(.185)	(.210)
Open Primary State	.20*	.09	.11	.17
	(.102)	(.119)	(.145)	(.172)
Partially Open Primary State	-.52***	-.39***	-.36***	-.42***
	(.092)	(.081)	(.099)	(.126)
Age	.06***	.03***	.05***	.04***
	(.005)	(.005)	(.006)	(.005)
Age Squared	-.0002***	-.0001***	-.0002***	-.0001**
	(.00004)	(.00004)	(.00005)	(.00004)
Female	.08***	.02	.03***	.04**
	(.016)	(.013)	(.010)	(.014)
Asian	-.45***	-.61***	-.81***	-.81***
	(.106)	(.087)	(.111)	(.081)
Black	-.60***	-.42***	-.21***	-.06
	(.056)	(.058)	(.080)	(.094)
Latino	-.77***	-.75***	-.60***	-.51***
	(.074)	(.057)	(.058)	(.040)
Race Other	-.47***	-.42***	-.41***	-.35***

	(.065)	(.041)	(.048)	(.051)
Married	.09**	.21***	.10***	.04
	(.035)	(.015)	(.033)	(.037)
Pr(Bachelor's degree)	-.003***	.003***	.003***	.01***
	(.001)	(.001)	(.001)	(.001)
Political Ideology	.02***	.01***	.002	.001
	(.002)	(.002)	(.002)	(.002)
Dem. Senate Fract. 2022	.24	.44	.63*	.76*
	(.291)	(.326)	(.345)	(.411)
Dem. Governor Fract. 2022	-.17	-.17	-.20	-.11
	(.136)	(.180)	(.205)	(.225)
Rep. Senate Fract. 2022	.27	.28	.31	.44
	(.188)	(.222)	(.250)	(.268)
Rep. Governor Fract. 2022	.14	.37*	.51**	.54**
	(.171)	(.195)	(.237)	(.266)
COVI 2020	.06	.03	.02	.03
	(.059)	(.052)	(.061)	(.066)
Constant	-6.26***	-4.25***	-4.14***	-4.15***
	(.233)	(.160)	(.234)	(.262)
Observations	413,361	1,370,491	300167.00	278357.00
Log-likelihood	-94788.22	-531194.72	-144309.94	-143809.57
Pseudo R2	.27	.23	.22	.20
BIC	189835.08	1062672	288872.11	287869.87

Catalist voter file data 2023. Unstandardized logistic regression coefficients. Standard errors are clustered by state in parentheses. $^{*}p < .01$, $^{**}p < .05$, $^{***}p < .001$

Appendix Table A3: Probability of voting in the 2022 primaries, lagging turnout in the 2018 primaries, nationwide for education group terciles (low, medium, high), 1% sample voter file

	(1)	(2)	(3)
	**Lowest Third Education**	**Middle Third Education**	**Highest Third Education**
Vote in 2018 Primary	2.43***	2.05***	1.87***
	(.032)	(.028)	(.034)
Non-partisan Primary State	.70***	.99***	1.14***
	(.152)	(.188)	(.205)
Open Primary State	.08	.12	.22
	(.112)	(.148)	(.160)
Partially Open Primary State	-.41***	-.41***	-.38***
	(.073)	(.104)	(.146)
Age	.03***	.05***	.03***
	(.006)	(.003)	(.008)
Age Squared	-.0001	-.0002***	-.0001
	(.00004)	(.00003)	(.0001)
Female	.03**	.05***	.06***
	(.013)	(.012)	(.015)
Asian	-.64***	-.76***	-.73***
	(.102)	(.097)	(.060)
Black	-.37***	-.36***	-.31***
	(.069)	(.058)	(.037)
Latino	-.77***	-.55***	-.42***
	(.061)	(.037)	(.032)
Race Other	-.41***	-.42***	-.32***
	(.043)	(.044)	(.049)
Married	.08***	.13***	.06*
	(.022)	(.019)	(.034)
Income	.31***	.18***	.15***
	(.020)	(.016)	(.015)
Political Ideology	.01***	.01***	.01***
	(.002)	(.001)	(.002)
Dem. Senate Fract. 2022	.40	.55	.87*
	(.298)	(.367)	(.460)
Dem. Governor Fract. 2022	-.20	-.16	-.07
	(.168)	(.214)	(.208)
Rep. Senate Fract. 2022	.26	.37	.35
	(.220)	(.242)	(.243)

Rep. Governor Fract. 2022	.36*	.45*	.50**
	(.183)	(.240)	(.251)
COVI 2020	.02	.04	.04
	(.054)	(.057)	(.066)
Constant	-4.71***	-5.10***	-4.66***
	(.164)	(.170)	(.280)
Observations	1,376,422	769,283	216,671
Log-likelihood	-477131.4	-334671.08	-103182.18
Pseudo R2	.26	.23	.21
BIC	954545.50	669613.22	206610.08

Catalist voter file data 2023. Unstandardized logistic regression coefficients. Standard errors are clustered by state in parentheses. * $p < .01$, ** $p < .05$, *** $p < .001$

Appendix Table A4: Table A1 above Omitting Covariate for Electoral Competition

	(1)	(2)	(3)
	Ages 18-34	Ages 35-64	Ages 65+
Vote in 2018 Primary	1.95***	2.20***	2.69***
	(.057)	(.032)	(.054)
Non-partisan Primary State	.70***	.54***	.37**
	(.148)	(.164)	(.153)
Open Primary State	.07	.06	.11
	(.138)	(.143)	(.147)
Partially Open Primary State	-.26	-.29**	-.23
	(.166)	(.141)	(.161)
Female	.06***	.05***	.02
	(.022)	(.013)	(.012)
Asian	-.53***	-.73***	-.90***
	(.062)	(.087)	(.163)
Black	-.73***	-.30***	-.30***
	(.049)	(.067)	(.088)
Latino	-.76***	-.73***	-.91***
	(.053)	(.045)	(.067)
Race Other	-.43***	-.40***	-.57***
	(.043)	(.055)	(.083)
Married	.06	.07***	.15***
	(.036)	(.026)	(.026)
Pr(Bachelor's degree)	-.00*	-.00	.00*
	(.001)	(.001)	(.001)

Income	.34***	.27***	.20***
	(.028)	(.024)	(.022)
Political Ideology	.01***	.00**	.01***
	(.002)	(.002)	(.002)
COVI 2020	.03	.04	-.03
	(.082)	(.079)	(.073)
Constant	-3.90***	-2.98***	-2.71***
	(.169)	(.113)	(.111)
Observation	582988	1,164,623	765798
Log-likelihood	-155843.4	-461419.5	-<br>333761.21
Pseudo R2	.12	.19	.29
BIC	311885.94	923048.52	667725.65

Catalist voter file data 2023. Unstandardized logistic regression coefficients. Standard errors are clustered by state in parentheses. * $p < .01$, ** $p < .05$, *** $p < .001$

Appendix Table A5: Table A2 above Omitting Covariate for Electoral Competition

	Lowest Third of Catalist Education Variable	Middle Third of Catalist Education Variable	Highest Third of Catalist Education Variable
Vote in 2018 Primary	2.41***	2.04***	1.86***
	(.031)	(.031)	(.038)
Non-partisan Primary State	.45***	.63***	.70***
	(.151)	(.177)	(.176)
Open Primary State	.11	.09	.08
	(.128)	(.161)	(.184)
Partially Open Primary State	-.29**	-.25	-.21
	(.137)	(.159)	(.165)
Age	.03***	.05***	.03***
	(.006)	(.003)	(.008)
Age Squared	-.00	-.00***	-.00
	(.000)	(.000)	(.000)
Female	.03**	.04***	.05***
	(.013)	(.012)	(.014)
Asian	-.66***	-.77***	-.76***
	(.113)	(.110)	(.069)
Black	-.39***	-.37***	-.31***
	(.074)	(.068)	(.041)

Latino	-.82***	-.61***	-.50***
	(.058)	(.046)	(.055)
Race Other	-.43***	-.44***	-.35***
	(.055)	(.059)	(.060)
Married	.09***	.15***	.07
	(.028)	(.027)	(.044)
Income	.31***	.17***	.15***
	(.022)	(.019)	(.020)
Political Ideology	.01***	.01***	.01***
	(.002)	(.001)	(.002)
COVI 2020	.00	.04	.09
	(.071)	(.083)	(.089)
Constant	-4.47***	-4.70***	-4.12***
	(.189)	(.157)	(.230)
Observations	1,376,422	769,283	216,671
Log-likelihood	-479518.57	-337431.64	-104289.42
Pseudo R2	.26	.22	.21
BIC	959263.31	675080.14	208775.42

Catalist voter file data 2023. Unstandardized logistic regression coefficients. Standard errors are clustered by state in parentheses. * $p < .01$, ** $p < .05$, *** $p < .001$

Appendix Table A6: Table A3 above Omitting Covariate for Electoral Competition

	(1)	(2)	(3)	(4)
	Income Less than $30,000	Income Between $30,000 and $75,000	Income Between $75,000 and $100,000	Income Above $100,000
Vote in 2018 Primary	2.43***	2.26***	2.11***	1.97***
	(.042)	(.029)	(.037)	(.040)
Non-partisan Primary State	.59***	.56***	.58***	.59***
	(.159)	(.149)	(.186)	(.196)
Open Primary State	.23**	.11	.08	.03
	(.106)	(.133)	(.167)	(.196)
Partially Open Primary State	-.40***	-.26*	-.22	-.24
	(.122)	(.135)	(.171)	(.185)
Age	.06***	.03***	.05***	.04***
	(.005)	(.005)	(.006)	(.005)
Age Squared	-.00***	-.00***	-.00***	-.00

	(.000)	(.000)	(.000)	(.000)
Female	.07***	.02	.03***	.03**
	(.016)	(.012)	(.010)	(.015)
Asian	-.47***	-.63***	-.83***	-.83***
	(.116)	(.098)	(.122)	(.097)
Black	-.61***	-.44***	-.25***	-.07
	(.062)	(.065)	(.086)	(.106)
Latino	-.82***	-.81***	-.67***	-.59***
	(.084)	(.054)	(.053)	(.065)
Race Other	-.49***	-.44***	-.44***	-.37***
	(.075)	(.052)	(.063)	(.071)
Married	.09**	.22***	.12***	.06
	(.038)	(.019)	(.042)	(.040)
Pr(Bachelor's degree)	-.00***	.00***	.00**	.00***
	(.001)	(.001)	(.001)	(.001)
Political Ideology	.02***	.01***	.00	.00
	(.002)	(.002)	(.002)	(.002)
COVI 2020	.04	.01	.03	.06
	(.067)	(.073)	(.087)	(.091)
Constant	-6.12***	-3.96***	-3.73***	-3.54***
	(.243)	(.168)	(.191)	(.206)
Observations	413,361	1,370,491	300,167	278,357
Log-likelihood	-94993.48	-<br>534131.65	-<br>145666.87	-<br>145622.53
Pseudo R2	.27	.23	.21	.19
BIC	190193.88	1068489	291535.54	291445.64

Catalist voter file data 2023. Unstandardized logistic regression coefficients. Standard errors are clustered by state in parentheses. * $p < .01$, ** $p < .05$, *** $p < .001$

Appendix Table A7 below shows turnout in the 2022 primary election nationwide, broken down by demographic groups. The data in top part of this table are-generated only using Catalist individual data. The table illustrates the bias in turnout in primary elections compared to general elections by demographic groups.

Values in the second-to-last row are the aggregate primary and general turnout reported by the Bipartisan Policy Center (Ferrer and Thorning, 2023) and the United States Election Project (USEP). The Catalist estimates of overall turnout reported in the last row closely track the aggregate primary turnout reported by the PBC.

Appendix Table A7: 2022 Primary and General Election Turnout by Demographic Groups

	Voted 2022 General Election	Voted 2022 Primary Election
Age: Below 25	26.64	8.14
Age: 25-39	31.98	10.16
Age: 40-54	44.26	16.25
Age: 55-72	56.92	28.85
Age 73+	57.59	25.23
Male	45.41	19.19
Female	45.25	19.54
College Degree	61.80	25.96
No College Degree	40.84	17.62
Income: Less than $20,000	13.32	5.08
$20,000 - $29,999	23.07	9.27
$30,000 - $49,999	37.32	15.23
$50,000 - $74.999	51.34	22.46
$75,000 - $100,000	61.99	27.91
$100,000 - $149,999	71.77	31.50
$150,000+	82.02	35.60
*Overall aggregate VAP turnout from the US Election Project & Bipartisan Policy Center	43.00	20.2
Overall turnout from individual voter file	*42.88*	*19.38*

Catalist 1% sample voter file data 2023

Introduction

We merged the 2022 Cornell Collaborative Midterm Survey (CMS)[1] with the January 2023 Catalist[2] voter file data of over 265 million US adults. The CMS responses were collected between October 26, 2022 and November 25, 2022 and funded by the National Science Foundation (NSF). Because a subsample of the CMS provided their names and addresses, we matched a subset of the CMS respondents to their personal voter file records. Of the 3500 people in the CMS contacted by IPSOS asked for identifying information, 2165 respondents were willing to provide their names and addresses, which could then be merged with their scores in the 2022 Catalist voter file.

The below crosstabs show the share of people who identify as female, as male, and as other on both CMS and in Catalist. This approach is used for family income as well.

Over 97% of cases have the same gender in both Catalist and CMS. Gender in Catalist is from the state voter files. The 3 percent could include people who self-identify as a different gender then their biological gender.

Table A8: Comparing Survey and Administrative Data Gender

		Gender Catalist (Administrative)		
		Female	Male	Other
Gender Self-Reported CMS	Female	1,255<br>(97.21%)	23<br>(2.71%)	13<br>(52%)
	Male	32<br>(2.48%)	823<br>(97.05%)	9<br>(36%)
	Other	4<br>(0.31%)	2<br>(0.24%)	3<br>(12%)

Note: Percentages are based on column frequencies.

Self-reported income can vary from credit bureau reports of family income in terms of whether the respondent is considering pre or post-tax income, varying time frames, W2 income or side job income (1099, untaxed), or individual or family income. Table 2 should be read down the columns. But the categories are generally comparable when considering proximate income categories. For people earning less than 20K a year, 81% self-report income of less than 20K or 20-50K categories. For people earning 20-50K in the credit bureau reports, 25% report less than 20K income, and 60% self-report incomes 20-50K or 50- 100 K. For people earning 100-150K in the credit bureau data, 83% self-report incomes in the categories of 50-100K, 100-150K or 150K or higher. There is generally a high match when adjacent categories are considered. While credit bureau income may differ modestly from self-reports, it is widely used in the economics literature and is a valid measure of household income.

[1] 2022 Collaborative Midterm Survey, Cornell Center for the Social Sciences. https://ropercenter.cornell.edu/2022-collaborative-midterm-survey

[2] https://catalist.us/data/

Table A9: Comparing Survey and Administrative Data Income

		Income Catalist (Credit Bureau Reports)				
		Less than $20K	$20K-$50K	$50K-$100K	$100K-$150K	$150K+
Income Self Reported CMS	Less than $20K	46<br>(48.94%)	293<br>(24.73%)	58<br>(8.19%)	1<br>(.60%)	0<br>(0%)
	$20K-$50K	30<br>(31.91%)	431<br>(36.37%)	176<br>(24.86%)	17<br>(10.24%)	0<br>(0%)
	$50K-$100K	10<br>(10.64%)	284<br>(23.97%)	252<br>(35.59%)	46<br>(27.71%)	2<br>(16.6%)
	$100K-$150K	4<br>(4.26%)	78<br>(6.58%)	126<br>(17.80%)	41<br>(24.70%)	4<br>(33.3%)
	$150K+	1<br>(1.06%)	52<br>(4.39%)	71<br>(10.03%)	49<br>(29.52%)	5<br>(41.7%)
	Prefer not to say	3<br>(3.19%)	45<br>(3.80%)	25<br>(3.53%)	12<br>(7.23%)	1<br>(8.3%)
	Total	100%	100%	100%	100%	100%

Note: Cells display number of observations; percentages are based on column frequencies.
